# Estimating the Cost of Delivering Tobacco Cessation Intervention Package at Noncommunicable Disease Clinics in Two Districts of North India

**DOI:** 10.1093/ntr/ntad105

**Published:** 2023-07-04

**Authors:** Garima Bhatt, Sonu Goel, Tanvi Kiran, Sandeep Grover, Bikash Medhi, Gurmandeep Singh, Sandeep Singh Gill

**Affiliations:** Department of Community Medicine and School of Public Health, Post Graduate Institute of Medical Education and Research (PGIMER), Chandigarh-160012, India; Department of Community Medicine and School of Public Health, Post Graduate Institute of Medical Education and Research (PGIMER), Chandigarh-160012, India; Faculty of Education & Health Sciences, University of Limerick, Ireland; Honorary Professor in the Faculty of Human & Health Sciences at Swansea University, United Kingdom; Department of Community Medicine and School of Public Health, Post Graduate Institute of Medical Education and Research (PGIMER), Chandigarh-160012, India; Department of Psychiatry, Postgraduate Institute of Medical Education and Research (PGIMER), Chandigarh, India; Department of Pharmacology, Postgraduate Institute of Medical Education and Research (PGIMER), Chandigarh, India; National Health Mission, Department of Health & Family Welfare Government of Punjab, Chandigarh, India; National Programme for Prevention & Control of Cancer, Diabetes, Cardiovascular Diseases & Stroke (NPCDCS), Department of Health & Family Welfare, Government of Punjab, Chandigarh, India

## Abstract

**Introduction:**

Integrated care is likely to improve outcomes in strained healthcare systems while limiting costs. NCD clinics were introduced under the “National Programme for Prevention and Control of Cancer, Diabetes, Cardiovascular Disease, and Stroke” (NPCDCS) in India; however, there is limited literature on the costs of delivering tobacco cessation interventions within NPCDCS. One of the study’s objectives was to estimate the cost of delivering a culturally specific patient-centric behavioral intervention package in two district-level NCD clinics in Punjab, India.

**Methods:**

Costing was undertaken using the health systems perspective. A top-down or financial costing approach and a bottom-up or activity-based approach were employed at each step of development and implementation. The opportunity cost was used to include the cost of human resources, infrastructure, and capital resources used. All infrastructure and capital costs were annualized using a 3% annual discount rate. Four additional scenarios were built up concerning three major components to reduce costs further when rolled out on a large scale.

**Results:**

The cost of intervention package development, human resource training, and unit cost of implementation were estimated to be INR 6,47,827 (USD 8,874); INR 134,002 (USD 1810); and INR 272 (USD 3.67), respectively. Based on our sensitivity analysis results, the service delivery cost varied from INR 184 (USD 2.48) to INR 326 (USD 4.40) per patient.

**Conclusion:**

The development costs of the intervention package accounted for the majority proportion of the total cost. Of the total unit cost of implementation, the telephonic follow-up, human resources, and capital resources were the major contributory components.

**Implications:**

The current study aims to fill gaps by estimating the unit-level health systems cost of a culturally sensitive, disease-specific, and patient-centric tobacco cessation intervention package delivered at the outpatient settings of NCD clinics at the secondary level hospital, which represents a major link in the health care system of India. Findings from this study could be used to provide supportive evidence to policymakers and program managers for rolling out such interventions in established NCD clinics through the NPCDCS program of the Indian Government.

## Background

The World Health Organization (WHO) indicates that if well-timed interventions to prevent and control Noncommunicable Diseases (NCDs) are not implemented, the total annual death toll from NCDs will increase to 55 million by 2030.^1^ Tobacco use is an important risk factor for NCDs and accounts for 14% of all NCD deaths in persons aged 30 and above worldwide.^2^ In 2017–2018, the overall financial burden from tobacco use equaled more than 1% of India’s total Gross Domestic Production. In addition, the annual direct healthcare expenditure for managing tobacco-related diseases accounted for 5.3% of India’s total health expenditures.^3^

Target 3.4 of the SDG Goal 3 (Good health and well-being) aims to reduce premature mortality from NCDs by one-third by 2030. Cessation is fundamental to achieving these development targets^.4,5^ However, ensuring that the interventions focused on tobacco cessation adequately reach the targeted population is still a major challenge. At present, about 30% of total tobacco users have adequate access to required tobacco cessation services.^6^ There is ample evidence of effective tobacco cessation programs in reducing NCDs in the short term and curbing healthcare costs.^7^

The WHO- Global Investment Case for Tobacco Cessation has estimated an investment of US$1.68 per capita over ten years (2021–2030) in cessation interventions like toll-free quit-lines, advice by healthcare staff in primary care, and m-cessation programs that can result in quitting successfully by 152 million users.^8^ India is a party to WHO-Framework Convention on Tobacco Control (FCTC). It is making efforts to implement Article 14 in tandem with the “O” (Offer help to quit tobacco use) component of the MPOWER policy package.^9,10^ Meeting international and national health goals depend on investments in tobacco cessation programs to increase the reach to tobacco users worldwide.

The Indian Government introduced the “National Programme for Prevention and Control of Cancer, Diabetes, Cardiovascular Disease, and Stroke” (NPCDCS) in 2010 to bolster the early diagnosis and management of NCDs.^11^ One of the key functions of these NCD clinics is to screen patients for risk factors, including tobacco use, and promote health by counseling users to quit.^12^ However, there is limited literature on integrating tobacco cessation interventions within NCD control.^13,14^ A systematic literature review and meta-analysis reported that integrated tobacco cessation with routine healthcare services would likely decrease costs and yield better health outcomes in strained healthcare systems by delivering high-quality services.^15^ It has been documented that integrated approaches between programs (HIV-TB, TB-DM) are more cost-effective than vertical approaches.^16,17^ In contrast, the literature on the cost of integrating the NCD control program with tobacco control is minimal, especially from the Low & Middle-Income Countries (LMICs).^18,19^

A systematic review of cost evaluation studies of tobacco control programs in clinical settings reported the importance of capturing the delivery cost to optimize monetary allocation to tobacco addiction management.^20^ Few studies have estimated the cost of tobacco cessation interventions, but they are limited by their contexts (most of them are in developed nations),^20^ comprehensibility (have undertaken fewer cost components only),^21^ and settings (most in hospital settings).^22,23^ A full economic analysis of a hospital-based initiated cessation program at Ontario, Canada, concluded that the cost of subsequent management of ailments arising from continuous smoking was significantly higher as compared to the intervention costs to stop smoking.^22^ Another systematic review suggests that economic analyses to evaluate resource allocation in tobacco cessation and control programs in clinical settings has been scarce over time.^20^ A study in Vietnam using community level smoking cessation intervention estimated overall costs during the development phase and the implementation, excluding opportunity costs.^21^ Another study conducted in the Pediatric emergency department of Midwestern Children’s Hospital examined the costs associated with implementing smoking cessation interventions and not its development.^23^

It is thus important that cost analyses of such programs are routinely undertaken and conveyed in simple language for non-experts to explore and collate the opinion of key stakeholders. Furthermore, research that uses empirical costing analysis approaches and incorporates an organization’s perspectives is highly effective.^24^ The information benefits the health systems developing such programs in the planning phase to implement these programs.^20^ Within this context, the objective of the current study was to undertake cost estimates for delivering a culture-specific, patient-centric behavioral intervention package for the tobacco users attending the NCD clinics.

### Ethics statement:

The Institute Ethics Committee (IEC) of the Post Graduate Institute of Medical Education and Research (PGIMER), Chandigarh, India (IEC number. INT/IEC/2017/1361) granted ethical approval. Prior permissions were obtained from the State Tobacco Control Cell and the NCD Control Cell, Department of Health & Family Welfare, Government of Punjab, India. The main study’s protocol has been registered with India’s Clinical Trials Registry, with the registration number CTRI/2018/01/011643.

## Methods

### Study Settings

The study was undertaken in two NCD clinics running at district-level facilities (with a population of 994,628 and 600,163), respectively, under the National Programme for Prevention and Control of Cancer, Diabetes, Cardiovascular Disease, and Stroke (NPCDCS) of Punjab, India. A total of 200 patients who were suffering from any NCD and were using tobacco were selected to implement the developed intervention package. They were followed up for 12 months.^25^ The intervention development activities were carried out in a phased manner from 2017 to 2018. Trainings for Health Care Providers were carried out in 2019, and intervention implementation began from April 2019 onwards. District Hospital is a hospital at the secondary referral level responsible for a defined geographical area for providing comprehensive secondary healthcare services.^26^ These clinics have dedicated staff (medical officer, counselor, nurse, data entry operator) to provide services for early diagnosis, treatment, follow-up for common NCDs, and screening for risk factors.^11^

### Costing Approach

The health systems perspective was used to estimate the costs; that is, all the costs incurred by the healthcare system in the development and its implementation were accounted for to reach the overall cost. We utilized a mixed-methods approach^27^ (using top-down and bottom-up costing methods) for calculating the cost of the tobacco cessation intervention package delivered at the NCD clinics. To estimate the intervention development cost, we employed a top-down or financial costing approach^28^ (i.e., the total cost of resources used to develop the intervention was summarized using administrative data). In contrast, the bottom-up or activity-based approach^29^ (identifying all resources and their quantity that will be used in the implementation process) of healthcare costing was used to estimate the cost of implementation of the package. We then applied monetary value (using actual cost or opportunity cost) to every resource according to the quantity used at every step.

Using the concept of opportunity cost, we included the cost of human resources utilized to develop and deliver the services, infrastructure used in the processes, capital resources like furniture and equipment utilized, cost of IEC material, cost of refreshments or stationery, cost of sending messages and making calls to patients, cost of travel and allowances to the experts, healthcare officials, participants and researcher in our analysis to make it comprehensive.

### Data Collection and Sources

**Figure 1.**
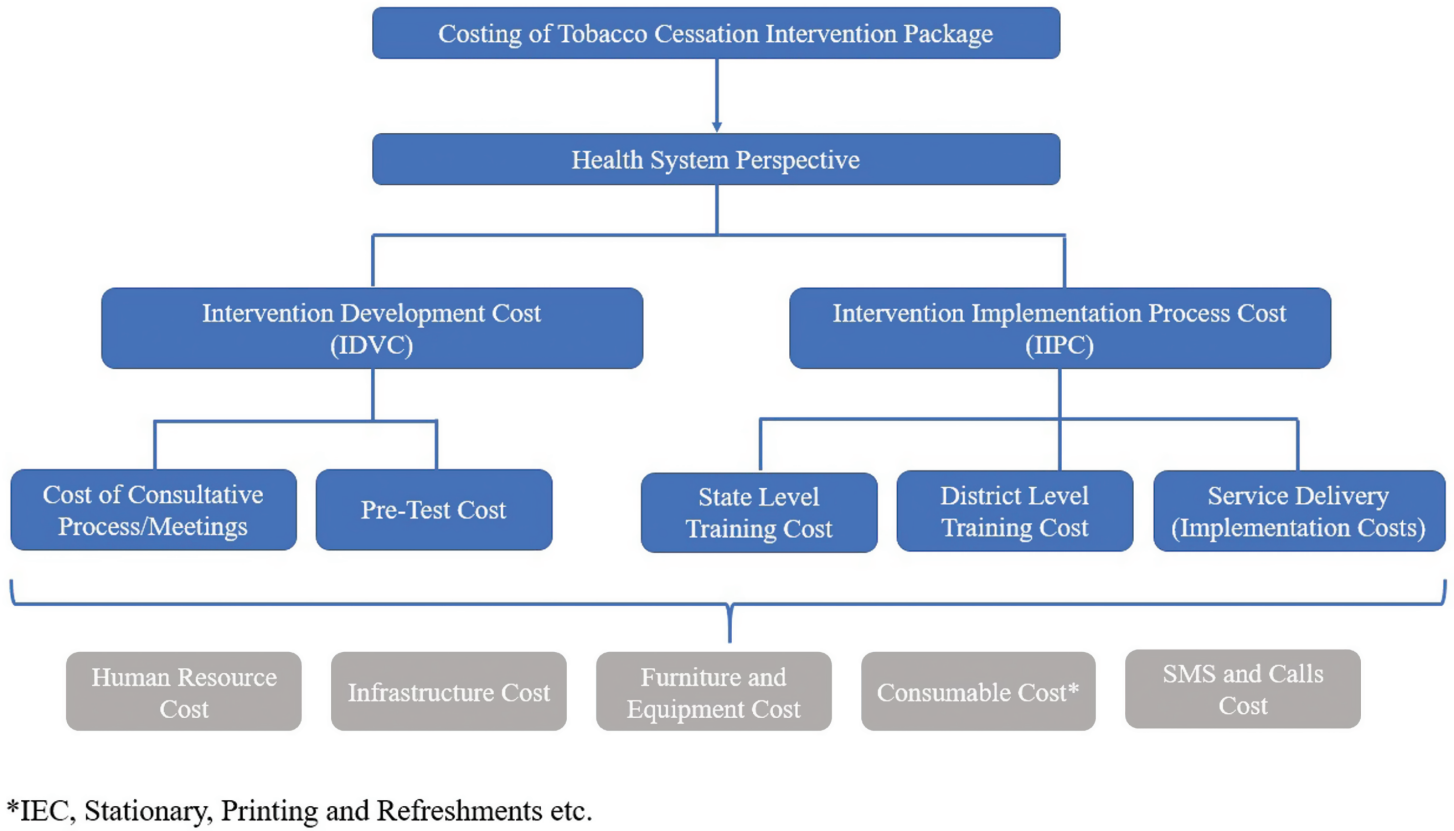
Conceptual Framework of the Cost Analysis.

We used a pragmatic approach (i.e., considering the feasibility aspect based on real-world program implementation settings) to collect data based on the availability and accuracy of the source to make our results close to reality. Information regarding the net and gross salaries of clinicians, government officials, healthcare staff, and other participants was collected from publicly available online documents and key informant interviews. Costs of venue employed during the design and implementation of the intervention were estimated using the rental prices of similarly built space in the region by surveying real-estate agents and enquiring about approximate rental value per square foot. The cost of overheads (stationary, travel, allowances, refreshments, and consumables) was estimated as per actuals/based on bills available. To estimate the cost of reminders to participants through calls and messages, we used BSNL tariff rates,^30^ available publicly on the internet. The current cost of other capital resources like furniture items and equipment used by the staff was taken from the online GeM portal of the Government of India,^31^ which was discounted, annualized, and then apportioned. The overall conceptual framework for the cost analysis is summarized below in Figure 1.

We retrospectively used the real record and bills to estimate the cost of meetings convened with experts from the health systems and tobacco control research community during the design of the intervention. Since most of these participants were from health systems and civil societies, we also incorporated the cost of their time, that is, opportunity cost based on their salary grades and allowances, which were obtained through key informant interviews. Then, the cost of trainers’ time and fee (in the form of honorarium offered to them per government norms), travel, and other expenditures like training material and IEC material were included as per record and bills. During service delivery, the total number of sessions required by the counselors, medical officers, nurses, and the researcher was identified, and the monetary cost was assigned to it using the concept of opportunity cost based on salaries and other allowances received by them by the government and time dedicated by them per patient. A summary of data collection and sources is given in Table 1.

**Table 1:** Various types of costs with the data sources and methodology)

Resource use^*^	Type of cost	Cost head	Type of data collected	Source of data	Assumptions (If any)	Method of data collection
IDVC & IIPC	Variable	Human Resource	Gross salaries	Staff Interviews and online government documents	Salaries of one cadre with similar years of service would be the same	Involved staff was interviewed, and data gaps were addressed by using online sources
IDVC & IIPC	Fixed	Infrastructure	Rental Cost	Key Informant Interviews	-	Primary data collection using the concept of opportunity cost
IIPC	Fixed	Furniture	Procurement Cost	GeM Portal and Government Procurement Tenders available online	Prices of the same quality equipment or furniture item used without being specific to the brands being used actually	Data collection from the Internet
IDVC & IIPC	Variable	Consumables	Procurement Cost	GeM Portal and Government Procurement Tenders available online	Prices of the same quality consumable item used without being specific to the brands being used actually	Data collection from the Internet
IDVC	Variable	Refreshment	Contract Rates	Actual billing data	-	Actual bills of contract rates were collected from vendors.
IDVC& IIPC	Variable	Traveling ­Allowance	Actual allowance paid/entitled	Staff Interviews and online government documents	The traveling allowance of one cadre with similar years of service would be the same.	Involved experts were interviewed, and data gaps were addressed using online sources.
IDVC	Variable	Honorarium to Experts	Actual honorarium paid/entitled	Staff Interviews and online government documents	An honorarium of one cadre with similar years of service would be the same.	Involved experts were interviewed, and data gaps were addressed using online sources.
IIPC	Fixed	SMS & Telephonic Reminders	Telephone bills	Tariff rates	Healthcare professionals would use BSNL (government enterprise)	Online tariff rates of BSNL for an appropriate package, including SMS and calling services, were used as a reference

^*^IDVC = Intervention Development Cost.

^*^IIPC = Intervention Implementation Process Cost.

### Data Analysis

We used Microsoft Excel to summarize and analyze the cost data for developing and implementing the intervention. We annualized all infrastructure and capital costs by using their average life expectancy (e.g., if an office table would last for approximately seven years, its cost was annualized over the period of seven years using an annualization formula and a 3% annual discount rate).^32^ We annualized all infrastructure and capital costs by using their average life expectancy (e.g., if an office table would last for approximately seven years, its cost was annualized over the period of seven years using an annualization formula and a 3% annual discount rate). The estimated annual cost was then changed into cost per minute and was used to estimate the cost of delivering the intervention to the patients. Likewise, the opportunity cost of human resources was estimated based on their gross salaries and the time given per patient to deliver the service. Since the implementation of the intervention would be spread over a period of time and many capital resources are to be used during the service delivery, we also discounted the cost of capital resources as suggested by various method manuals and guidance documents.^32,33^ Discounting is done to adjust the capital costs and expenditure over a longer period to the present value because of time preference to ensure comparability and standardization across costing evaluations. As suggested by the Health Technology Assessment in India (HTAIn) reference case, we have used a 3% annual discount rate.^32^

A one-way uncertainty analysis was run to address the variation in the implementation process and associated costs using the upper and lower bound of the resource costs and the quantity of resources required. In case the upper and lower limit of resource cost was not applicable, we varied the resource inputs by 20% on both sides in our uncertainty analysis. Based on these variations, the lowest and highest possible cost estimates to deliver the service to a patient were ascertained. We also ranked the top five most influential parameters that varied the overall cost and arranged them in their decreasing order using a tornado diagram. Besides, based on the expert opinion from the program managers, we also estimated the cost per patient in different scenarios if the intervention is scaled up for cost efficiency. We created the following four scenarios:

**Scenario 1: Disease-specific group counseling sessions.**
**Scenario 2: Bulk printing of disease-specific pamphlets and tailored short message service packages.**
**Scenario 3: Integrated sessions during routine in-service training programs for human resources.**
**Scenario 4: A cumulative discount scenario including group counseling and bulk procurement.**

We also undertook a budgetary impact analysis to estimate the overall cost incurred by one such clinic for initial and refresher trainings of the staff and the cost of implementation. We used national tobacco control program operational guidelines^34^ for the frequency of state and district-level trainings that staff should undergo for efficient program implementation. Also, at the current levels of capacity utilization, we estimated the overall cost of implementation annually for the next ten years for an indication of the program managers for better decision making and is provided in the Supplementary Materials.

## Results

The intervention development cost was estimated to be INR 6,47,827 (USD 8,874), including the cost of meetings, workshops, and advocacy programs. The cost of testing the preliminary format of the intervention was estimated to be INR 11,620 (USD 159), which comprises the cost of in-depth interviews of officers from different cadres, including medical officers, nurses, counselors, program officers, and tobacco users. The total cost of delivery of the intervention was estimated to be INR 134,002 (USD 1810), based on actual bills, including the opportunity cost of participants’ time, logistics, allowances, and other expenditures like honorarium, food, and refreshments. The unit cost of implementation was estimated to be INR 272 (USD 3.67), out of which telephonic follow-up, human resources, and capital resources were the major components. The overall results of the analysis are summarized in Table 2.

**Table 2:** Total costs of developing and delivering tobacco cessation intervention package at NCD clinic in two districts of North India.

Cost Components of Intervention	Cost INR (USD)	Proportional Cost (%)
**Intervention Development Cost (IDVC)**		
Meetings with civil society (n = 2)	9,396 (128)	1.42
Workshop with Program Manager (n = 1)	4,91,040 (6,726)	74.46
Meeting with Program Manager (n = 1)	93,795 (1,284)	14.22
Advocacy Workshop (n = 1)	53,593 (734)	8.13
Cost of doing a pre-test	11,620 (157)	1.77
Total	6,59,447 (8911)	100
**Unit Cost (IDVC)**	6,594 (89)	100
**Intervention Implementation Process Cost (IIPC)**		
Total Health System Cost of Training of HCP	1,34,002 (1810)	100
Unit Cost of Training of HCP	1340 (18.10)	100
Unit Cost of Service Delivery		
1. Human Resource	76 (1.02)	27.94
2. Capital Cost (Building, Furniture, Equipment)	26 (0.35)	9.56
3. Pamphlets (IEC)	10 (0.14)	3.68
4. SMS and Follow-up calls (HR)	160 (2.16)	58.82
5. Dedicated Phone Bill	0.15 (0.0019)	0.06
Overall Cost	272 (3.67)	100

**Figure 2.**
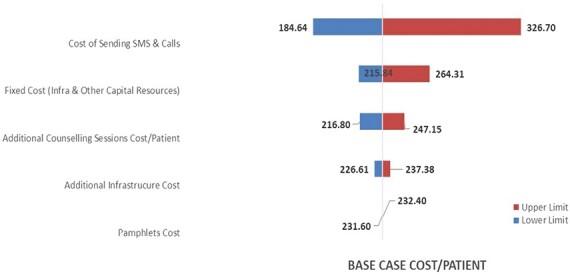
Tornado diagram illustrating uncertainty analysis of various input costs of tobacco cessation package.

Based on our sensitivity analysis results, the service delivery cost varied from INR 184 (USD 2.48) to INR 326 (USD 4.40) per patient. From the uncertainty analysis, in the decreasing order of its impact, the variance due to the overall cost of the package, cost of sending SMS & calls, fixed cost of capital resources, additional counseling session cost per patient, additional infrastructure cost, and pamphlet cost is summarized in the tornado diagram (Figure 2).

Based on assumptions guided by the expert opinion of the program managers, cost variance based on different levels and modes of program implementation was estimated. The cost of delivery in these four scenarios is elaborated in Table 3.

**Table 3.** Scenario building to reduce the cost of implementation

Scenario I	Impact on the delivery cost by adopting disease-specific group counseling sessions			
Base Case	Cost of Delivery of Service = INR 272			
Patient Group Size	Group of 5	Group of 10	Group of 15	Group of 20
Delivery cost per patient	INR 190	INR 180	INR 177	INR 175
**Scenario II**	Impact on delivery cost through bulk purchasing of pamphlets, calls, and SMS			
**Base Case**	Cost of Delivery of Service = INR 272			
Discount Scenario	At a 10 % Discount	At 20 % Discount	At 30 % Discount	At a 40 % Discount
Implementation cost per patient	INR 255	INR 238	INR 221	INR 204
**Scenario III**	Impact on training cost by integrating the training with currently ongoing programs			
**Base Case**	Total Health System Cost of Training of Healthcare Provider (HCP) = INR 1340			
Discount Scenario	At a 10 % Discount	At 20 % Discount	At 30 % Discount	At a 40 % Discount
Training cost per patient	INR 1206	INR 1072	INR 938	INR 804
**Cumulative Variance**	Minimal Discount Scenario (Groups of 5 each and 10% discount)		Optimistic Discount Scenario (Group of 20 each and 40% discount)	
Discounted cost of Implementation	INR 173		INR 107	
Total Discounted Cost of Training and Implementation	INR 1379		INR 911	

## Discussion

The current study’s findings were estimated based on the cost data for the development and implementation/delivery of a culturally sensitive, disease-specific, and patient-centric tobacco cessation intervention package collected from the outpatient settings of NCD clinics at two district-level facilities in North India. The multimodal intervention package was developed through the involvement of multi-stakeholders with consideration of the socio-cultural background of tobacco users and a patient-centric approach enhancing its acceptability. The face-to-face counseling sessions, pamphlets, and short text messages were tailored to tobacco users’ disease and stage of behavior change in the regional language, increasing its suitability to the context.

The outpatient settings of NCD clinics under the NPCDCS, Government of India, are an important locus for tobacco cessation. Every visit to the NCD clinics represents an opportune teachable moment for HCPs to screen & offer cessation support to NCD patients.^14^ The current study aims to fill gaps by estimating the unit-level health systems cost of a tobacco cessation intervention package delivered at the secondary-level hospital, representing a major link in the health care system of India.

The benefits of implementing tobacco cessation interventions in resource-constrained contexts are usually substantial, but these costs should be considered in economic evaluations. We followed a structured approach while considering costs incurred at each stage while undertaking the costing process. There are several strengths of the study. *First,* the study provides comprehensive cost estimates for developing and implementing a tobacco cessation intervention package at NCD clinics. *Second,* the costing approach was based on something other than budgetary information but comprehensively considered all the resources utilized and activities included during the development and implementation phases. *Third,* we undertook an uncertainty analysis and included opportunity costs. *Fourth,* we used both top-down and bottom-up approaches during our costing approach.

The Ministry of Health, Government of India, has advocated collaborations between national health programs wherein joint activities can be planned and carried out within the existing health system framework.^34^ The National Tobacco Control Program also emphasizes expanding the scope and quality of implementation of their tobacco cessation services at different levels of the Indian continuum of care to make the service delivery most efficient and cost-effective using available resources.^34^ Given that tobacco use is a modifiable and preventable behavioral risk factor for NCDs, NPCDCS anticipates fostering synergies with existing tobacco control programs. The cost information could benefit the healthcare system to plan, implement, and sustain new tobacco cessation interventions.^20^

The study provides the cost of development, pretest, and intervention implementation. The findings look largely consistent in pattern with pre-existent costing evidence globally, which estimated the cost of similar healthcare interventions to address tobacco addiction.^35,36^ The major component of this overall expenditure is incurred in the meetings and workshops with healthcare officials, similar to other tobacco control interventions globally.^37,38^ The package development cost [INR 6,47,827 (USD 8,874)] shared most of the cost component. The existing literature on estimating the cost of the planning and implementation of similar public health interventions in such resource-constraint geographies also reports that the design phase cost makes a significant proportion and usually the majority of the total cost of implementing the intervention.^39^ For instance, in a trial conducted in Uganda, where customized software was needed to deliver short messaging service (SMS) reminders to support the home-based TB screening program, the total cost to design and refine the SMS delivery intervention (estimated to be USD137/screened contact, and USD 90 went into software development) makes a significant amount of the total cost of intervention implementation (USD 54 per screened contact).^40^ Another study on cost analysis of the development of an intervention to increase uptake of diabetic retinopathy screening reported that the total cost of intervention development was €40,485, of which about 78% accounted for the human resource cost (€31,451).^41^ The costs of the intervention development phase are usually higher because it needs significant input in terms of time, human resources, and research both clinically as well as in the administrative setting. This time of human resources that goes into the development process has an opportunity cost. With already resource constraints in healthcare for research, capturing the actual cost of the intervention development and additional cost of implementation when the intervention is incorporated into already running programs can inform priorities for resource allocation and help policymakers.

In our analysis, although the cost of development of this intervention was significantly more than the cost of intervention delivery, this initial higher investment for the development and design of our intervention will eventually be counterbalanced by the scale-up of the intervention on a larger population. The delivery cost may vary considerably if the intervention is implemented across different states or regions based on various sites (human resources, infrastructure). The variation in the cost needs to be acknowledged by researchers who plan to develop similar interventions that can be more suitable to their local context and by decision-makers who plan to implement the intervention and would require a tailored budgetary impact analysis of the local healthcare infrastructure. Failure of these adjustments to understand the variance in the cost estimates of implementation and even the designing of similar interventions can result in significant underestimation or overestimation and could be problematic for implementers subsequently.^39^

During the implementation phase, another significant cost incurred was training the healthcare professionals [INR 1,34,002 (USD 1810)], which would help effectively deliver the intervention. During the implementation, a significant proportion of the unit cost is attributed to telephonic follow-up, human resources, logistics, and capital resources. This was validated by sensitivity analysis and again showed a similar pattern to the international literature.^42,43^ Rationale for this consonance in the resource utilization pattern is largely due to similarity in the overall nature of tobacco-cessation interventions through counseling and monitoring.^20^

The current study reported that the total incremental cost per patient (including human resource and capital costs) was INR 102.68 (USD 1.40). Given the high footfall of a public sector hospital in India and the limited time devoted to each patient, we propose delivering disease-specific (e.g., Diabetic/Hypertensive) group (8–10 patients) counseling sessions and treating these as single units. The counselor could deliver a group session (12–15 minutes) to the formed group(s) on dedicated days a week. Besides, NICE guidelines also suggest that group therapy is generally more cost-effective as the cost of therapist time is shared, and fewer trained professionals are required to provide treatment to more patients.^44^ However, evidence on disease-specific group counseling sessions for tobacco users remains minimal, especially in outpatient department settings.^45^

In the current study, the costs for disease-specific pamphlets, tailored SMS & follow-up calls incurred to be INR 10 ($0.14) and INR 160 ($2.16), respectively. These costs could be reduced through bulk purchases via bid proposals by getting the most qualified sellers of services while keeping costs low. Besides, bulk SMS service could allow the senders to instantly reach a large number of users, increase speed and allow for delivery of more tailored messages with lower operational costs. A study conducted in Sudan reported that although the respondents felt positive about receiving SMS but had concerns about privacy issues surrounding mobile advertising.^46^

The current study estimated the health system cost of HCP training to be INR 1,34,002 (USD 1810). The HCPs need to be trained before introducing a new intervention. Therefore, to reduce the costs associated with conducting exclusive training, sessions could be integrated within the existing schedule of training programs of various national health programs (NPCDCS, tuberculosis, oral health, maternal & child health, and mental health programs).^14^ A systematic review and meta-analysis on the cost and effects of integrated care reported that integrated care is likely to reduce cost and improve outcomes.^15^ These integrated sessions could be a part of trainings proposed in the Programme Implementation Plan (PIP) under the National Health Mission, Government of India.

When newly developed interventions are implemented and gradually scaled up, some initial costs, like training of the human resource, development of training materials, and infrastructure for the launch of implementation, are significant and are incurred up-front. Such initial fixed costs remain the same with the levels at which the service is planned to be implemented. The contribution of these up-front costs to the total implementation cost also can differ significantly depending upon various parameters like operational capacity, infrastructure, and overall implementation outcomes.^47^ The current study provides estimates for the “above-service” as well as service level healthcare costs of implementation of the intervention package, including salaries, capital costs, and consumables, thus reflecting the pragmatic (i.e., considering feasibility aspect, based on real-world program implementation settings) estimates of unit costs. However, these costs may vary from one site to another.^39^

### Limitations

There are certain limitations of the study which should be considered while interpreting the results. *First*, the study reports costs from a health systems perspective only. From a societal standpoint, the costs incurred by patients (e.g., patient opportunity costs for time) could be incorporated to get a realistic picture of the overall costs, including out-of-pocket expenditures. However, since we tried to synchronize the counseling sessions of the patients with their routine consultative follow-ups, it could have reduced the patient-related costs. *Second*, a complete economic evaluation could have been undertaken to demonstrate the value of the intervention package. We assessed the cost in two district-level facilities in one state, making it difficult to generalize the results at a national level. However, because a district is representative of most of the other districts of India and secondary healthcare of the country (in terms of resource availability & utilization, type of services delivered, and population catered), the findings could be indicative. *Third,* several resources used for intervention delivery were available as shared resources, so we apportioned the quantity explicitly used for the intervention package using apportioning statistics for resource use, as suggested in the costing literature,^48^ which may lead to some inaccuracy in estimates. *Fourth,* a time-motion study could have been better used to assess better the time of staff involved in multiple tasks and better understand the time allocation patterns and cost estimates.^48^ We undertook personal interviews with healthcare providers in both settings instead of time-motion studies to understand the time given to activities pertaining to our intervention and allocation of resources as our interview-based method is a comparatively easy and less costly way to capture time spent on relevant activities.^49^

## Conclusion

The overall cost of development and unit cost of implementation of the intervention package at two NCD clinics was estimated to be INR 6,47,827 (USD 8,874) and INR 272 (USD 3.67), respectively. Our estimates on cost are useful for policymakers and program managers to plan and implement tobacco cessation interventions in NCD settings. As healthcare is a state subject in India, we recommend estimating state-level cost estimates for the tobacco cessation package. The key factors that may influence the cost of delivery by states include diversity in human resources, the effectiveness of delivery, and accessibility of intervention, thus may not be generalizable. More evidence is needed regarding this strategy’s cost-effectiveness to demonstrate the package’s value. A few uncertainties, including politico-bureaucratic commitments, could not be accounted for in our analysis. To provide an honest appraisal of effectiveness, costs, and budgetary impact to the decision-makers; it is important to document input resources and cost of the expenses of intervention design, refinement, implementation, and scaling up to a larger population of interest, especially in resource-constrained settings. Literature suggests that continued tobacco use affects NCD progression and prognosis. Therefore, providing tobacco cessation interventions in the NCD clinic to a high-risk & receptive group could save costs of tobacco-induced diseases, prevent costs from its treatment, and improve the quality of life.

## Supplementary Material

A Contributorship Form detailing each author’s specific involvement with this content, as well as any supplementary data, are available online at https://academic.oup.com/ntr.

ntad105_suppl_Supplementary_Materials

## Data Availability

The data supporting this study’s findings are available on request from the corresponding author, [SG]. The data are not publicly available due to [restrictions, e.g., their containing information that could compromise the privacy of research participants].
