# Supplementary material for: Estimating the Cost of Delivering Tobacco Cessation Intervention Package at Noncommunicable Disease Clinics in Two Districts of North India
Source: Nicotine Tob Res. 2023 Jul 4;25(11):1727–35. doi: 10.1093/ntr/ntad105 (PMC10475607; doi:10.1093/ntr/ntad105)
Supplement: ntad105_suppl_Supplementary_Materials [file ntad105_suppl_supplementary_materials.zip › ntad105_suppl_Supplementary_Tables.docx]

**Supplementary table 1. Major heads of cost inputs and their classification**

| **Variable Costs** | **Fixed Costs** |
| --- | --- |
| - Human Resource (Salaries and Allowances) - Travel Cost (specialist and researcher) - Information, Education & Communication Material - Stationary (During development phase) - Food / Refreshments (Experts during meetings) - Cost of Telephonic Calls/ SMS to patients | - Building (Infrastructure used to deliver services) - Capital Resources (Equipment and Furniture) |

**Supplementary table 2: Detailed cost description of intervention development (IDVC) and intervention implementation (IIPC)**

| **Intervention Development Cost (IDVC)** | | | | | | | | |
| --- | --- | --- | --- | --- | --- | --- | --- | --- |
| Intervention development Process | | | | Estimated Cost (INR) | | Estimated Cost (USD) | | Percentage Share (%) |
| Meetings with civil society | | | | 9396 | | 128.71 | | 1.45 |
| Workshop with Program Manager | | | | 491040 | | 6726.58 | | 75.80 |
| Meeting with Program Manager | | | | 93795 | | 1284.86 | | 14.48 |
| Advocacy Workshop | | | | 53593 | | 734.15 | | 8.27 |
| TOTAL | | | | 6,47,827 | | 8874.34 | | 100 |
| **Cost of doing pre-test** | | | | | | | | |
| In-depth Interviews | | | | Total Cost(INR) | | Total Cost (USD) | | Percentage Share (%) |
| Medical officers | | | | 1,284 | | 17.59 | | 11.05 |
| Nursing Officers | | | | 1,049 | | 14.37 | | 9.03 |
| Counsellors | | | | 1,047 | | 14.34 | | 9.01 |
| Public Health Experts | | | | 1,284 | | 17.59 | | 11.05 |
| Tobacco Users | | | | 700 | | 9.59 | | 6.02 |
| Program Officers | | | | 1,355 | | 18.56 | | 11.66 |
| Researcher's Time (Opportunity Cost) | | | | 4,900 | | 67.12 | | 42.17 |
| GRAND TOTAL | | | | 11,620 | | 159.18 | | 100 |
| **Intervention Implementation Process Cost (IIPC)** | | | | | | | | |
| **Cost of Training of HCP (Two District Level Workshops)** | | | | | | | | |
| Expenditure Head | | | | Overall Cost (INR) | | Cost (USD) | | Share (%) |
| Opportunity Cost of Participants | | | | 7102 | | 95.97 | | 51.12 |
| Master Trainers | | | | 298 | | 4.03 | | 2.14 |
| Travelling Allowance | | | | 200 | | 2.70 | | 1.44 |
| Tea/Lunch | | | | 150 | | 2.03 | | 1.08 |
| Printing And Stationary | | | | 5520 | | 74.59 | | 39.73 |
| Institutional Overhead | | | | 468 | | 6.32 | | 3.37 |
| Floor Area (Venue Cost) | | | | 153 | | 2.07 | | 1.10 |
| Total (INR) | | | | 13,893 | | 187.74 | | 100 |
| **Cost of Training of HCP (State)** | | | | | | | | |
| Expenditure Head | | | | Overall Cost (INR) | | Cost (USD) | | Share (%) |
| Tea Snacks and Tentage | | | | 41005 | | 554.12 | | 34.14 |
| Stationary | | | | 2537 | | 34.28 | | 2.11 |
| Flex and Banner | | | | 2962 | | 40.03 | | 2.47 |
| Booklets | | | | 27600 | | 372.97 | | 22.98 |
| Honorarium to Trainers | | | | 10000 | | 135.14 | | 8.33 |
| Travelling Allowance | | | | 17500 | | 236.49 | | 14.57 |
| Opportunity Cost of Participants | | | | 14750 | | 199.32 | | 12.28 |
| Floor Area (Venue Cost) | | | | 3755.45 | | 50.75 | | 3.13 |
| Total (INR) | | | | 1,20,109 | | 1623.09 | | 100.00 |
| **Unit Cost of Service Delivery** | | | | | | | | |
|  | Opportunity cost per minutes | Time per Patient in Control Group (mins) | | | Time per Patient in Intervention Group (mins) | | Cost Control  Group | Cost Intervention Group |
| Medical officer (Session I)  (2 minutes per patient) | 11.36 | 2 | | | 2 | | 22.73 | 22.73 |
| Medical officer (Session II)  (2 minutes per patient) | 11.36 | 2 | | | 2 | | 22.73 | 22.73 |
| Medical officer (Session III)  (2 minutes per patient) | 11.36 | 2 | | | 2 | | 22.73 | 22.73 |
| Medical officer (Session IV)  (2 minutes per patient) | 11.36 | 2 | | | 2 | | 22.73 | 22.73 |
| Counsellor (Session I)  (12 minutes per patient) | 1.89 | 2 | | | 12 | | 3.79 | 22.73 |
| Counsellor (Session II)  (12 minutes per patient) | 1.89 | 2 | | | 12 | | 3.79 | 22.73 |
| Counsellor (Session III)  (12 minutes per patient) | 1.89 | 2 | | | 12 | | 3.79 | 22.73 |
| Counsellor (Session IV)  (12 minutes per patient) | 1.89 | 2 | | | 12 | | 3.79 | 22.73 |
| Nurse (Session I)  (2 minutes per patient) | 1.97 | 2 | | | 2 | | 3.95 | 3.95 |
| Nurse (Session II)  (2 minutes per patient) | 1.97 | 2 | | | 2 | | 3.95 | 3.95 |
| Nurse (Session III)  (2 minutes per patient) | 1.97 | 2 | | | 2 | | 3.95 | 3.95 |
| Nurse (Session IV)  (2 minutes per patient) | 1.97 | 2 | | | 2 | | 3.95 | 3.95 |
| Capital Cost per Patient (Including Opportunity Cost of Building, Furniture, Equipment etc.) | | | Cost (INR) | | | Cost (USD) | | |
| Doctor’s Office (Per Minute Cost) | | | 0.93 | | | 0.012 | | |
|  | | |  | | |  | | |
| Counsellor’s Office (Per Minute Cost) | | | 0.67 | | | 0.009 | | |
| Total Incremental Cost/Patient (Including HR Cost and Capital Costs) | | | 102.68 | | | 1.40 | | |
| **Cost of implementation of the package by the researcher** | | | | | | | | |
| Component of intervention package | | | | Cost (INR) | | Cost (USD) | | Share (%) |
| Pamphlets | | | | 10.00 | | 0.14 | | 0.94 |
| SMS (opportunity cost of time of researcher) | | | | 79.55 | | 1.09 | | 7.32 |
| Follow up calls (opportunity cost of time of researcher) | | | | 159.09 | | 2.18 | | 14.63 |
| Dedicated phone bill | | | | 0.13 | | 0.00 | | 0.00 |
| Travel cost of researcher to district 1 per visit | | | | 240.00 | | 3.29 | | 22.08 |
| Travel cost of researcher to district 2 per visit | | | | 600.00 | | 8.22 | | 55.17 |
| Total Intervention cost per patient | | | | 1,088 | | 14.90 | | 100 |

Supplementary Table 3. Average life expectancy of furniture and equipment, as used in the analysis.

| Equipment and Furniture Item | Expected life of Equipment |
| --- | --- |
| Patient Stool | 7 |
| Office Chair | 7 |
| Ceiling Fan | 7 |
| AC | 7 |
| Cupboard (Big) | 10 |

Supplementary Table 4: Budget Impact Analysis of 10 years including Training and Implementation Cost

| **Budget Impact Analysis** | Annual Cost | 10 Years Cost |
| --- | --- | --- |
| Training Cost |  |  |
| Staff to be Trained |  |  |
| Medical Officer | 1 | |
| Nursing Officer | 2 | |
| Counsellor | 1 | |
| Total Staff | 4 | |
| Training Cost for District Level  (4 Trainings every Year) | 5538 | 55378 |
| Training Cost for State Level Training (1 Training every Year) | 12011 | 120109 |
| **Total Training Cost** | 17549 | 175488 |
| Implementation Cost |  |  |
| Number of Patients Catered | 8890 | 88900 |
| **Total Cost of Implementation** | 2418080 | 24180800 |
|  |  |  |
| Overall Cost Per Clinic | 2435629 | 24356288 |
