# Supplementary figures and images for: Estimating the Cost of Delivering Tobacco Cessation Intervention Package at Noncommunicable Disease Clinics in Two Districts of North India
Source: Nicotine Tob Res. 2023 Jul 4;25(11):1727–35. doi: 10.1093/ntr/ntad105 (PMC10475607; doi:10.1093/ntr/ntad105)

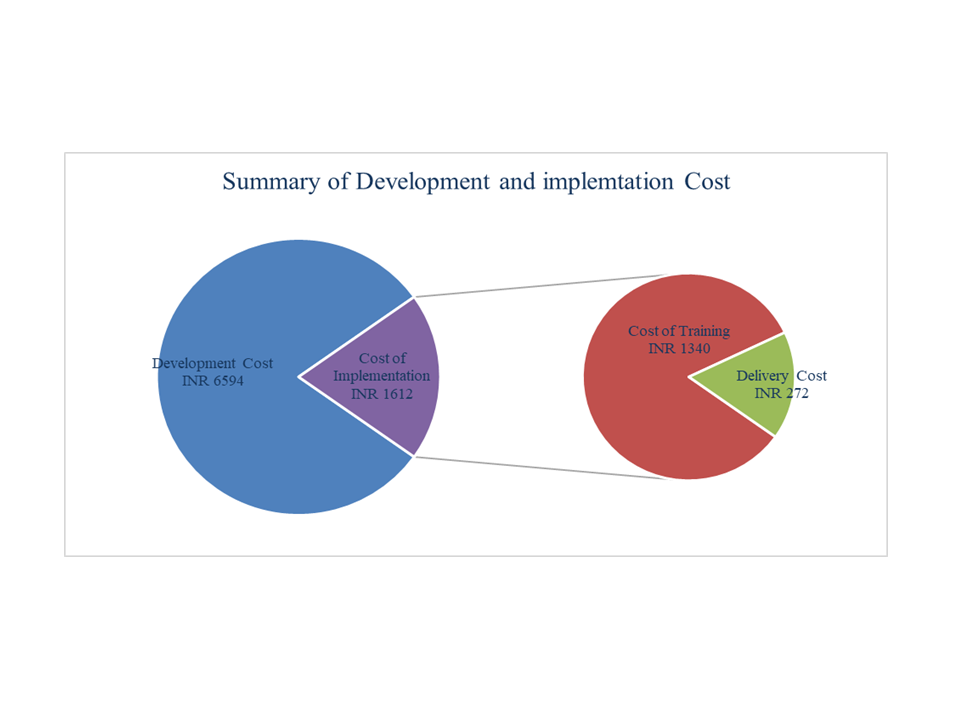

Supplement: ntad105_suppl_Supplementary_Materials [file ntad105_suppl_supplementary_materials.zip › ntad105_suppl_Supplementary_Figure_S1.tif]
